# Intention to Screen for Hepatitis C Among University Students: Influence of Different Communicative Scenarios

**DOI:** 10.3389/fpsyt.2022.873566

**Published:** 2022-05-11

**Authors:** Pierluigi Diotaiuti, Stefania Mancone, Lavinia Falese, Maria Ferrara, Fernando Bellizzi, Giuseppe Valente, Stefano Corrado, Francesco Misiti

**Affiliations:** Department of Human Sciences, Society and Health, University of Cassino and Southern Lazio, Cassino, Italy

**Keywords:** hepatitis C, risk perception, early detection intention, narrative scenarios, gender identification, effective communication, university students

## Abstract

This study aimed to evaluate the influence of different narrative scenarios regarding students' intentions to undergo diagnostic screening for hepatitis C, and whether gender identification with the characters of the scenario could influence the students' intentions to undergo a medical test. A sample of 600 participants was administered three narrative scenarios with different frames (positive, negative, and ambivalent), including two gender options (male and female) for the main character of the story. A statistically significant three-way interaction between scenario, gender identification, and time resulted. There were significant simple main effects on the intention to have a diagnostic test for hepatitis C for the scenarios with the protagonist of the same gender as the participant and after the administration of the negative scenario. The use of a negative scenario with the same gender character was always more effective than the use of a positive framed scenario, even though there was a high level of knowledge regarding the disease. Personal diagnostic testing was not directly associated with knowledge regarding the infection. The findings of this study can ultimately help policymakers develop communication campaigns adapted to target populations such as college students, in order to raise awareness of the risk, promote prevention and behavioral change, and encourage medical screening.

## Introduction

Hepatitis C is a major health problem that consists of the inflammation of the liver caused by hepatitis C virus (HCV) infection. HCV can have both acute and chronic consequences, and it is one of the leading causes of cirrhosis and hepatocellular carcinoma (1, 2). New HCV infections are usually asymptomatic, or the symptoms can take up to 30 years to appear (3). The consequence of this gap is that approximately 70% of infected people are not aware that they are infected, so they do not follow therapies or treatments, and the transmission takes place very easily (4, 5).

Hepatitis C is present throughout the world, with a high prevalence in European countries. The World Health Organization (WHO) estimates 58 million people are affected by chronic HCV and 290,000 deaths in 2019 (6). Italy appears to be one of the European countries with the highest number of patients with hepatitis C. Epidemiological data show an estimated prevalence of the chronic disease in about 3% of the population (1.5 million people), with a very variable geographical distribution. The frequency of infection is higher in the center and south of Italy than in the north (7, 8). A study promoted by Doxa Pharma, “Italians and Hepatitis C,” has highlighted citizens' lack of knowledge regarding this disease. The study found that 69% of respondents knew little or nothing about hepatitis C and tended to underestimate its severity (9).

Today, the major risk factors for getting infected are surgery, blood transfusions, organ transplants, percutaneous exposure during cosmetic treatments, unprotected sexual relations, and intravenous drug use (3). Piercing and tattoos are also risk factors for hepatitis C infection when these practices are performed with non-sterile instruments and in unsafe environments (10). Even manicures or pedicures performed with non-sterilized or disposable instruments represent a significant danger of infection. Many practices related to the transmission of the virus can be considered more common among young people. Scientific evidence shows that risky behaviors are widespread among young people and requires particular attention and adequate measures, not only because of the serious health and psycho-social implications but also because of the association with multiple risk behaviors (10, 11).

For this reason, we decided to carry out research on this specific age target, focusing on university students.

Health promotion among university students has not always been considered a priority in targeting preventive policies and actions because students are assumed to be in a relatively healthy phase of their life. However, this period is very critical, since it is known to be a dynamic transition from childhood to adulthood. During this time, young people should gradually learn to assume responsibility for their own health, but they often adopt unhealthy behaviors and habits (poor eating habits, little rest, physical inactivity, smoking, alcohol and drug abuse, and risky sexual behaviors) that can negatively affect health in the short, medium, and long term (12, 13). The university environment can stimulate students to assume risky lifestyles for various reasons: the first time away from families and their rules; personality traits; experience of rebel models or just adult models; and self-challenge. Unconstrained freedom mixed with a sense of invulnerability and a strong desire for exploration can lead to the development of behaviors that are not always healthy (14, 15).

Since there is no effective vaccination against the hepatitis C virus, information and health education represent the best preventive strategy (16). Increasing the level of awareness and knowledge can reduce the risk of transmission and lead to healthier behaviors (17, 18). Other studies have shown that the lack of knowledge and a low level of awareness are also big barriers to HCV screening (19, 20), and the early diagnosis can be the keystone in virus management (21). A group of experts interviewed to discuss relevant aspects and open issues of chronic hepatitis C in Italy and concluded that one of the main barriers to HCV care is the low screening rate (22).

Health and screening programmes often lack information regarding the benefits and harms of screening tests and do not favor the decision-making process by individuals (23). In the past, to achieve public health objectives and induce behavioral changes, traditional health communication mostly reported statistics and probabilities; however, recently, new forms of narrative communication are spreading, demonstrating efficacy if properly addressed (24).

Previous research examined the framing effect on individual choices in terms of prevention and health treatments, identifying different modalities such as the framing of attributes, objectives, and risky choices (25, 26).

The application of a narrative scenario approach has been used in sociology, communication, and marketing research (27, 28) and recently in health psychology as well (29–31).

Narrative scenarios create a simulation of a possible future that can be experienced as a realistic, consistent, and compelling plot, allowing the person to explore a possible reaction (32). According to Campi and Garatti (33), fictionalized scenarios are experiments conceived with a high degree of imagination and realism as they explore, in particular, the human and social dimensions in the setting of everyday life.

The purpose of this study was to assess the influence of different narrative scenarios on university students' intentions to undergo diagnostic testing for hepatitis C and to investigate whether gender identification also influences the outcomes.

### Hypotheses

a) The use in a preventive communication intervention of a narrative frame with different content orientation (positive, negative, and ambivalent) can affect the propensity (immediate, medium term, long term, and null) to perform a diagnostic test for hepatitis C.

b) Gender identification with the protagonists of each narrative frame can also influence the propensity to perform a diagnostic test.

## Methods

### Participants

The study was voluntarily attended by 600 university students in central Italy: 300 male (50%) and 300 female (50%); aged between 18 and 32; M_age_ = 25; and *SD* = 5.8. The inclusion criterion in the study allowed participation of all generic regular undergraduate adult students who aged 18 years and above, and who were present at the time of data collection. Students who gave consent for the study were recruited. The study participants who were blind and severely ill were excluded from the study. Participants were assured anonymity and the use of data in aggregate form for research purposes only. Tools administration took place upon release and signing of the form for informed consent of participation in accordance with the Declaration of Helsinki. Among all those who were willing to participate in the study, there were no drop-outs or incomplete deliveries of materials. Table 1 reports the characteristics of the participants.

**Table 1:** Characteristics of the participants.

Gender	Males = 300 Females = 300
Study course	Economy = 66 (11.0%) Foreign languages = 36 (6.0%) Pedagogical sciences = 72 (12.0%) Motor sciences = 54 (9.0%) Law = 90 (15.0%) Humanities = 54 (9.0%) Communication sciences = 30 (5.0%) Engineering = 108 (18.0%) Nursing sciences = 24 (4.0%) Social work = 66 (11.0%)
Year of course	First = 157 (26.1%) Second = 149 (24.8) Third = 116 (19.3%) Fourth = 92 (15.3%) Fifth = 65 (10.8%) Out-of-course = 21 (3.5%)
Father's education	Primary school = 32 (5.4%) Secondary school = 127 (21.1%) High school diploma = 276 (46.0%) University degree = 165 (27.5%)
Mother's education	Primary school = 45 (7.5%) Secondary school = 148 (24.6%) High school diploma = 264 (43.9%) University degree = 143 (24.0%)
If one or both parents work in health professions	Yes = 95 (15.8%) No = 505 (84.2%)

### Tools

1) A socio-personal data questionnaire and collection of participants' knowledge on hepatitis C;
2) Evaluation of the importance attributed to performing personal diagnostic screening measured by means of an item on a Likert scale 1–5 ranging from 1 (not at all) to 5 (very much);
3) Three narrative scenarios with different values (positive, negative, and ambivalent), including two gender options (male and female) in the textual character:
  - *Positive scenario*: e.g., “Francesca is a 25-year-old girl. Three years ago, following a check-up, she discovered she was suffering from hepatitis C. After the discovery of the disease she immediately started the course of treatment. Today Francesca is married, has a child and leads a normal and satisfying life. For Francesca the timeliness of the check-up and the beginning of the therapy were decisive in order to prevent the disease from becoming chronic and causing serious consequences to the liver system.”
  - *Negative scenario*: e.g., “Francesca is a 25-year-old girl who was referred to visit her family doctor following the appearance of worrying symptoms such as jaundice (yellowish complexion of the skin and eyes), nausea, vomiting and abdominal pain. The check-up revealed the existence of chronic hepatitis C. The onset of the infection dated back to 5 years before. During this time, Francesca did not undergo any tests. Today, despite having cured the infection, Francesca has suffered serious damage to her liver system, affecting the quality of life and life expectancy.”
  - *Ambivalent scenario*: e.g., “Marco is a 25-year-old boy. After a medical check-up he had a year ago, he discovered that he had hepatitis C. He went to a specialist and was informed about the existence of the latest cure using a drug called “sofosbuvir,” which eliminates the disease permanently without significant side effects. Unfortunately, the treatment is extremely expensive, about 60,000.00 Euros and is not provided by the national health service. At this point, Marco was forced to access older treatments, which have little effect and serious side effects.”
5) Evaluation of the propensity to undergo diagnostic screening, measured with multiple response items 1–6, ranging from 0 (“I have no intention to undergo screening”) to 6 (“I intend to undergo screening immediately”).

### Design

Participants (balanced by gender) were randomly assigned to different groups depending on the experimental condition. To verify the hypothesis that both the scenario used and the identification (based on gender) have some effect on the propensity for diagnostic screening, the groups which were given the scenario with the protagonist of the same gender as the participant were compared with the groups which were given the scenario with the protagonist of the opposite gender to the participant. Before and after the administration of the scenario, the participant was asked to estimate his or her intention to have a diagnostic check for hepatitis C. Therefore, the research involved a 3 ×2 ×2 model: a total of six groups with the distribution of three scenarios and two options of identification with the gender of the protagonist (same sex/opposite sex) (between-subjects factors) and a pre-post evaluation of the intention to have a diagnostic check-up (within factor). Table 2 shows the participants' randomized distribution among the groups.

**Table 2:** Research sample distribution.

**Category variable**	**Modalities**	**Sub-modalities**	**Randomized distribution**	**Relative size**
Gender	Male			300
	Female			300
	Positive	Male character	administered to 50 males and 50 females	100
		Female character	administered to 50 males and 50 females	100
Scenario	Negative	Male character	administered to 50 males and 50 females	100
		Female character	administered to 50 males and 50 females	100
	Ambivalent	Male character	administered to 50 males and 50 females	100
		Female character	administered to 50 males and 50 females	100
				Total *N* = 600

### Procedure

The protocol therefore provided in sequence:

1) Collection of personal data in anonymous form (gender, age, course of study, year of course, parents' level of education);
2) Administration of a form to detect (a) knowledge possessed by the participants regarding hepatitis C and potential risk behaviors; (b) estimate of the risk of personal infection; (c) national epidemiological estimate on hepatitis C; and (d) direct knowledge of cases of hepatitis C. Table 3 shows the list of requests in the form.
3) Assessment of personal intention to carry out a diagnostic control using an item (Likert 1–6) to measure the (timely or procrastinating) propensity of the participants to undergo a diagnostic test for hepatitis C, ranging from 0 (“I have no intention to undergo a check”) to 6 (“I intend to undergo a check immediately, within a week”). Table 4 shows the personal screening intention form.
4) Administration (33.3%, *N* = 200) of a form with the *Positive Therapeutic Scenario* (early detection of infection and early therapeutic treatment with effective results).
5) Administration (33.3%, *N* = 200) of a form with the *Negative Therapeutic Scenario* (description of treatments with heavy and ineffective side effects).
6) Administration (33.3%, *N* = 200) of a form with the *Ambivalent Scenario* (description of new drugs with high efficacy and low side effects, “sofosbuvir,” but not accessible due to high costs).
7) Administration (the whole sample) of the item (Likert 1–6) to remeasure (timely or procrastinating) the intention of the participants to undergo a diagnostic test for hepatitis C, ranging from 0 (“I have no intention to undergo a check”) to 6 (“I intend to undergo a check immediately, within a week”).

**Table 3:** Knowledge form about hepatitis C.

Have you ever been checked for hepatitis?	yes; no
Which hepatitis is more dangerous?	Hepatitis A; Hepatitis C
Hepatitis damages:	Heart; Pancreas; Liver; Kidneys; Lungs
Hepatitis C can be contracted through:	
- Sexual intercourse	yes; no
- Ingesting seafood or raw fish	yes; no
- Eating raw frozen berries	yes; no
- Cosmetic treatments	yes; no
- Blood transfusions	yes; no
- Contact with saliva and sweat	yes; no
- Drinking contaminated water	yes; no
- Piercing	yes; no
- Drinking alcohol and hard liquor	yes; no
- Hugs	
- Sneezing	
- Dental treatment	
- Tattooing	
- Swapping syringes	
- Snorting cocaine	
How many people do you think are affected by hepatitis C in Italy?	Approximately 30,000; Approximately 400,000; Approximately 1,000,000; Approximately 6,000,000
How likely is it for a university student to contract hepatitis C?	very unlikely; unlikely; probable; very probable; highly probable
Most affected by hepatitis C:	Men; Women
Are there any categories of people at higher risk of infection?	yes; no
If yes, which?	Indicate
How many people do you know who have hepatitis C?	None; at least one; more than one
What check(s) should be performed to detect hepatitis C?	
- Urine test	yes; no
- Blood test	yes; no
- Stool test	yes; no
- Liver biopsy	yes; no
- X-ray examination	yes; no
How important do you think it is for you to be checked for hepatitis?	very little; a little; somewhat; quite a lot; very much

**Table 4:** Diagnostic screening intention form.

The Department of Human Sciences, Society and Health would like to propose to the University that a service be made available to students for free and anonymous testing for the hepatitis C virus. To set up this service, students need to book in advance.
When would you like the check-up to take place?
- In one week - In one month - In three months - In six months - In nine months - I have no intention of carrying out any checks
I don't want to carry out the check at the presidium set up by the University, but I still intend to carry out a check privately:
- In one week - In one month - In three months - In six months - In nine months - I have no intention of carrying out any checks

### Statistical Analysis

The data were processed using the statistical software SPSS version 26. The main analyses performed were: descriptive statistics to illustrate socio-demographic information and participants' knowledge of hepatitis C; Pearson bivariate correlations between risk perception and value attributed to personal diagnostic screening, significant at *p* < 0.001, two-tailed; one-way ANOVA to assess whether the estimated spread of the disease could be significantly associated with the value attributed to personal screening. The verification of the assumptions of univariate normality has been conducted using the procedure for the standardization of the variables, by inspection of a boxplot, and using Shapiro-Wilk's normality test. The homogeneity of variances was assessed by Levene's test. To assess the effects between narrative scenario types and gender identification in the scenarios, a three-way repeated measures ANOVA was run with three independent variables (scenario × gender identification × time) and one dependent variable (intention to carry out a diagnostic test for hepatitis C). As the number of participants was balanced in the groups, in order to determine the interaction between the variables, Pillai's criterion rather than Wilks' lambda was used, as it is more robust to unequal covariance matrices (34). Following the study by Cohen (35), partial eta squared was used to assess effect size (0.01 = small, 0.06 = medium, and 0.13 = large). The level of significance was set at *p* < 0.05, while for testing the multiple univariate interaction effects, a Bonferroni adjustment has been introduced by dividing the declared level of statistical significance by the number of dependent variables: *p* < 0.025 (i.e., *p* < 0.05/2).

## Results

### Descriptive Analysis

In total, 28.6% of participants stated that they had already taken a hepatitis C test in the past; 14.7% had one or both parents working in healthcare; 14.7% knew at least one person with hepatitis C; and 5.5% said they knew two or more people with hepatitis C. Neither the level of education nor the profession of the parents had any influence on the importance attributed to undergoing a diagnostic test for hepatitis C. The assessment of the students' knowledge on hepatitis C was carried out by summing up the exact answers to the questionnaire. The analysis of the distribution of the answers indicated that out of 19 questions proposed, the average of the correct answers was 12.8 (*SD* = 2.61, minimum 4 and maximum 17, skewness: −0.392, and kurtosis: −0.207). About half of the sample (53.3%) showed that they had a low to medium level of general knowledge on hepatitis C.

Comparing the students' national estimated spread of hepatitis C with their value placed on medical screening, it was found that those who tended to underestimate the incidence of the infection, considering it in ~30,000 (M_1_) or ~400,000 (M_2_) cases estimated in Italy, had significantly lower averages of value attribution to medical checks, while those who indicated the real official estimation of ~1,000,000 (M_3_) in Italy or overestimated the prevalence with more than 6,000,000 (M_4_) cases, attributed greater value to personal medical checks: *F*(3,599) = 10.091; *p* = 0.001; η^2^ = 0.04; OP = 0.98; M_1_ = 3.43, *SD* = 0.07; M_2_ = 3.45, *SD* = 0.05; M_3_ = 3.78, *SD* = 0.05; M_4_ = 3.85, *SD* = 0.12; 95% CI [3.55, 3.70].

A slightly positive correlation was found (*r* = 0.162^**^) between the attribution of value on diagnostic screening and the probability of risk attributed to the category of university students (to which the participants of the study belonged). However, 38.9% believed that a university student becoming infected with hepatitis C is unlikely or completely improbable. When asked whether there are categories of people at higher risk of infection, 50.8% said yes, and Table 5 illustrates the categories indicated in the descending order of frequency.

**Table 5:** People believed to be at a higher risk of infection.

Drug addicts	86 (14.3 %)
Homosexuals	74 (12.3 %)
Prostitutes	68 (11.3 %)
People with low immunity	60 (10.0%)
Health care personnel	53 (8.8 %)
Patients requiring transfusions	35 (5.8 %)
People practicing unsafe sex	30 (5.0 %)
People with serious illnesses	26 (4.3 %)
The elderly	24 (4.0 %)
Alcoholics	21 (3.5 %)
People with an irregular lifestyle	21 (3.5 %)
People with poor hygiene	20 (3.3 %)
HIV positives	16 (2.7 %)
Pregnant women	16 (2.7 %)
People with a predisposition to the disease	15 (2.5 %)
People who travel a lot to high-risk countries	15 (2.5 %)
People with an irregular diet	10 (1.7 %)
People who are not vaccinated	10 (1.7 %)

Of note, 34.9% stated that men are more exposed to hepatitis C, 7.5% believed that women are more exposed, while 57.5% indicated that the risk affects both genders equally.

### The Effect of Narrative Scenarios and Gender Identification on the Propensity for Medical Screening

A three-way repeated measures ANOVA (3 × 2 × 2) was conducted to examine both the effects of scenarios and gender identification on the intention to have a diagnostic test for hepatitis C. Residual analysis was performed to test the assumptions of the three-way repeated measures ANOVA. Outliers were assessed by visual inspection of a boxplot. Normality was assessed using Shapiro-Wilk's normality test for each cell in the design, and homogeneity of variances was assessed by Levene's test. There were no outliers, residuals were normally distributed (*p* > 0.05), and there was homogeneity of variances (*p* = 0.859). A statistically significant three-way interaction between scenario, gender identification, and time resulted, *F* (1, 99) = 6.399, *p* < 0.01. Statistical significance was accepted at the *p* < 0.025 level for simple two-way interactions and simple main effects. There were statistically significant simple two-way interactions between gender identification and time, *F* (1, 299) = 18.141, *p* < 0.025; scenario and time, *F* (1, 199) = 14.056, *p* < 0.025; and scenario and gender identification, *F* (1, 99) = 4.270, *p* < 0.025.

As regards the first interaction, there was a statistically significant simple main effect on the intention to have a diagnostic test for hepatitis C for the scenarios with the protagonist of the same gender as the participant, *F* (1, 299) = 22.808, *p* < 0.025. A Bonferroni adjustment was applied. For the scenarios with the protagonist of the same gender as the participant, the mean intention to have a diagnostic test before the scenarios administration was 3.53 (*SE* = 0.51), while after the scenarios administration was 4.10 (*SE* = 0.11). There was a statistically significant mean difference in the intention to have a diagnostic test between the two moments of −0.566; 95% CI (−0.799, −0.333), *p* < 0.025, ηp^2^ = 0.067. For the scenarios with the protagonist of the opposite gender as the participant, the simple main effect between the two moments was non-significant: *p* = 0.515, i.e., > 0.025.

As regards the second interaction, there was a statistically significant simple main effect on the intention to have a diagnostic test for hepatitis C after the administration of the negative scenario, *F* (1, 199) = 76.305, *p* < 0.025. For participants inserted in the group with the negative scenario, the mean intention to have a diagnostic test before the scenario administration was 3.53 (*SE* = 0.50), while after the scenario administration was 4.47 (*SE* = 0.09). There was a statistically significant mean difference in the intention to have a diagnostic test between the two moments of −0.942; 95% CI [−1.15, −0.730], *p* < 0.025, ηp^2^ = 0.183. For the positive and the ambivalent scenarios, the simple main effect between the two moments was non-significant: *p* = 0.344 and *p* = 0.186, i.e., > 0.025.

As regards the third interaction, there was a statistically significant simple main effect on the intention to have a diagnostic test for hepatitis C after the administration of the negative scenario where there was identification with the gender of the protagonist, *F* (1, 99) = 25.712, *p* < 0.025. For this condition, the mean intention to have a diagnostic test before the scenario administration was 4.89 (*SE* = 0.12), while after the scenario administration was 3.63 (*SE* = 0.20). There was a statistically significant mean difference in the intention to have a diagnostic test between the two moments of 1.261; 95% CI [0.768, 1.75], *p* < 0.025, ηp^2^ = 0.189. For the positive and the ambivalent scenarios, the simple main effect related to the use of a same or an opposite gender identification was not significant: *p* = 0.061 and *p* = 0.644, i.e., > 0.025.

**Figure 1:**
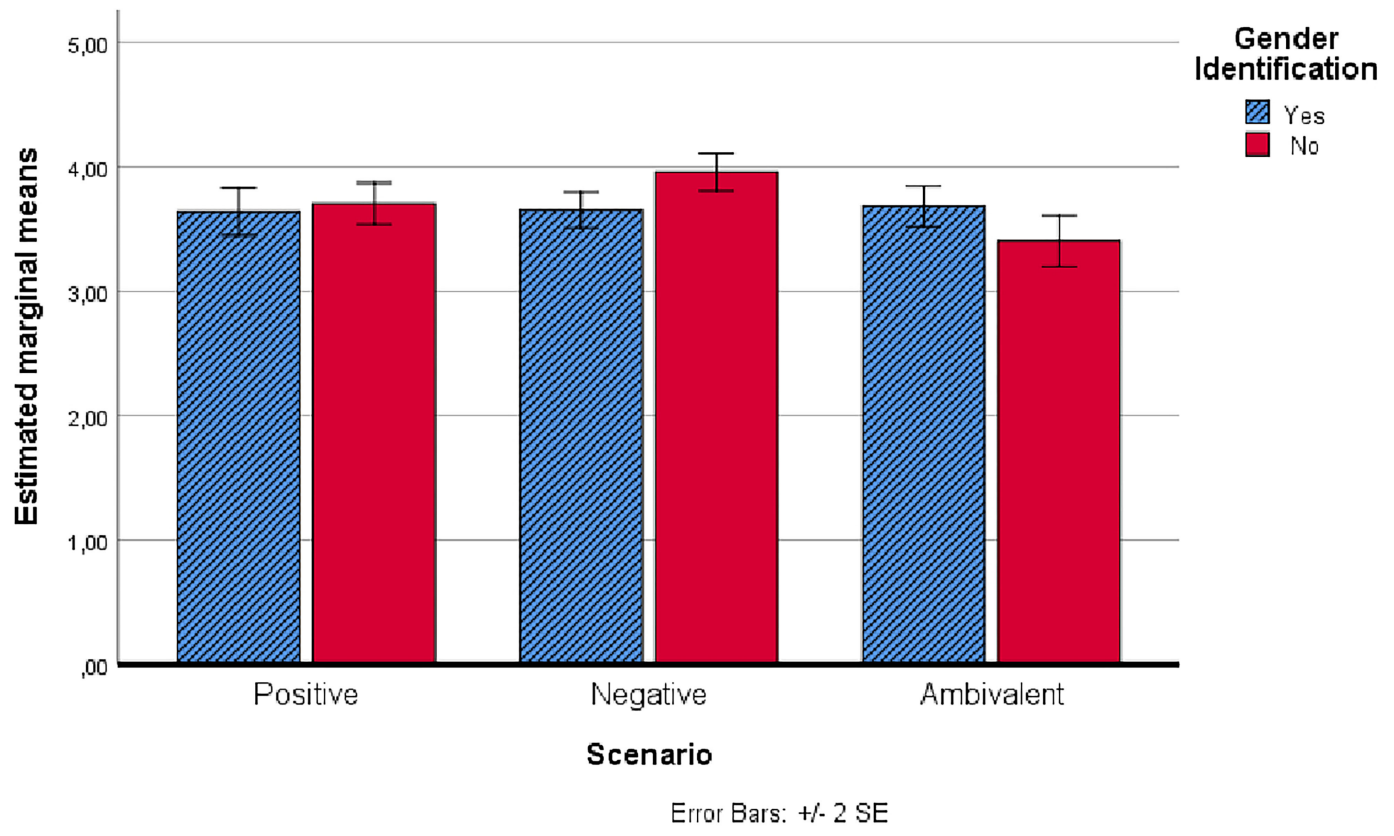
Estimated marginal means of the intention to carry out a diagnostic test for hepatitis C before the administration of scenarios.

**Figure 2:**
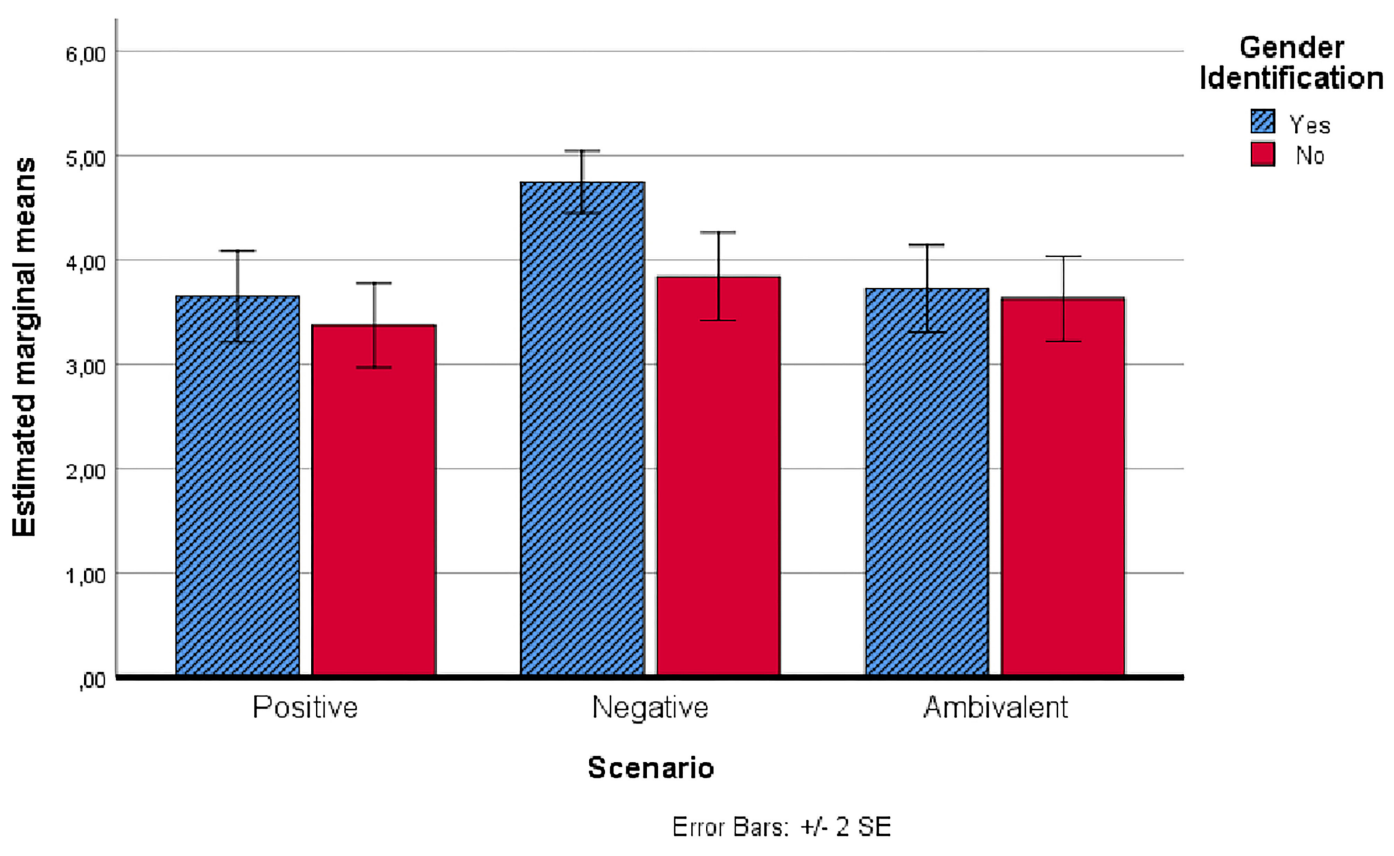
Estimated marginal means of the intention to carry out a diagnostic test for hepatitis C after the administration of scenarios.

Figures 1, 2 show the trends in the intention to carry out a diagnostic test for hepatitis C, before and after scenarios administration, also considering gender identification with the protagonist of the scenarios.

After the administration of the scenarios, it was found, as shown in Figure 2, that the intention to undergo a diagnostic screening was significantly higher in the group that had received the negative scenario with the character of the same gender as the participant.

## Discussion

For more than half of the sample in the study, the level of general knowledge about hepatitis C infection was medium to high, with only 9% of the sample providing <10 out of 19 correct answers to the questionnaire administered. This result is similar to what was found in the study by Daniali et al. (16), although in that case, the sample was made up of students from the medical-health area, where a greater average knowledge and awareness of the risks related to such infections was likely to be expected (16). A common result between our study and the one mentioned above was the absence of positive correlation between the level of knowledge and behavioral intention of prevention. The level of knowledge was not associated with the socio-cultural level of belonging, suggesting that there are other sources of information (presumably school, friends, direct, and indirect contact with infected people). In total, 30% of the sample had already undergone a hepatitis C test, and 20.2% had direct knowledge of people with hepatitis C. The importance given to personal diagnostic testing was not directly associated with the level of specific knowledge about the infection but was rather proportionate to the estimated spread of hepatitis C throughout the country. Those who tended to underestimate the spread of infection consequently attached less value to medical monitoring (31, 36).

However, we found an association between the importance attributed to diagnostic screening and the level of risk attributed to the category of university students (to which the participants belonged). These results appear consistent with Kasperson et al. (37).

With regard to the effect of the use of narrative scenarios on the propensity for diagnostic screening, it was found that the group presented with the negative scenario reported significantly higher levels of propensity for screening, especially compared to the group with the positive scenario. It was likely that the positive scenario had reassuring effects that limited the participants' propensity for screening: an early diagnosis was followed by an adequate course of treatment that allowed the protagonist to lead a substantially normal life; while the negative scenario increased the levels of fear: a late diagnosis was followed by a progressive worsening of health conditions and a heavy impact of drugs and their side effects on the protagonist's quality of life. In the ambivalent scenario, it was probably possible to activate simultaneously different emotional states, such as fear, anger, and resignation. The placement of the level of propensity for screening in an intermediate position with respect to the groups with positive and negative scenarios suggests that the activating effect of fear could have received moderation from any feelings of anger and resignation induced by the representation of such a scenario.

On the importance of eliciting emotions through the use of narratives to motivate changes in attitudes, intentions, and behaviors related to health issues, the contribution of Dunlop et al. (38) should certainly be considered. Self-referent emotional responses are expected to have a direct effect in motivating behavior change, particularly as they are likely to be associated with an increase in perceived personal risk. Message-referent and plot-referent emotional responses are proposed to have indirect effects on the individual, primarily by stimulating self-referent emotions, and prompting interpersonal discussion about the message. Hoeken and Sinkeldam (39) also pointed out that identification with a story character can evoke emotions that subsequently influence the audience's attitude. In their study, they reported on the mediating role of emotions in narrative persuasion and how identification can evoke these emotions. Liu and Yang (40), in a study using different frames of narrative persuasion to curb e-cigarette use among university students, reported that in a gain-framed narrative, the elicitation of a negative emotion such as anger was associated with an increasing risk perception and decreasing intention to use e-cigarettes. Some recent studies testing the effect of negative emotions such as fear, anger, and sadness in narrative persuasion interventions for health were conducted by Lillie et al. (41, 42), Jensen et al. (43), and Krakow et al. (44). They demonstrated that story outcome (e.g., whether the main character lives or dies) can impact audience behavior. Death narratives generated greater fear, anger, and sadness. Fear was related to greater behavioral intention and reading flow and diminished counterarguing. Sadness had the opposite effect. Anger produced a mixed persuasive effect, increasing both counterarguing and reading flow.

In our study, the positive and ambivalent scenarios, however, suggested that there is a cure and they probably lower the fear of the disease; with regard to the ambivalent scenario, we can hypothesize the stimulation of anger or resignation as the last emotion the person came into contact with, when the information passed on stated that the cure existed but was not available due to economic reasons. The recent study by Scherr et al. (45) showed that anger solicitation in the use of persuasive scenarios lowers the intention to follow the desired directions of the preventive campaign.

Compared to the research hypothesis, we would have expected that scenarios would play a greater role in stimulating the idea of undergoing diagnostic investigation to facilitate early diagnosis. In this case, we hypothesized that the third-person scenario had a reassuring or defensive role for the participant, since a change of perspective probably occurs by moving from a subjective self-referencing point of view to an external hetero referent point of view. Indeed, this observation is consistent with the review by De Graaf et al. (30), where they state that a first-person perspective is a promising feature in terms of effectiveness. The results of our study showed that, at least in the youth population, even if the level of awareness of the disease is medium-high, the propensity for diagnostic screening is positively associated with the use of a negative scenario, which leverages fear as a motivating emotion (46, 47).

The results also showed that in addition to the use of a negative scenario, the presence of a character of the same gender as the participant leads to a more effective outcome in terms of intention to screen. When scenarios do not present a possibility of gender identification, they may be less effective. The propensity to be more involved with characters, whose gender role is similar to one's own, has already been reported in several previous studies (48–53).

As highlighted by Metwally et al. (54), in addition to raising awareness about the disease and risk behavior for hepatitis C infection, communication campaigns should focus on promoting appropriate behavior and changing potentially dangerous practices. Therefore, the main result of our study shows that the use of a communication strategy that employs specific narrative scenarios and exploits the principle of gender identification can increase early diagnostic screening behavior, which especially among young people, given the extended incubation interval of the disease, is of fundamental importance to intervene promptly with pharmacological administration to contain further spread of the virus.

A further strength of the study is the awareness-raising intervention for university students with no medical background. This age group is particularly exposed to experimentation, unruliness, promiscuity, and sometimes superficiality in assessing the medium- and long-term consequences of their actions (55–57).

Another element that emerged in the study is the presence of stereotyped beliefs or prejudices that lead young learners to believe that risk concerns specific categories and therefore they are convinced that they are not affected by the problem. Effective educational interventions should aim at dismantling such misconceptions and evaluative distortions in order to induce a more cautious and responsible attitude.

If we consider the data relating to direct knowledge of at least one infected with hepatitis C, 14.7%, added to the 5.5% of those who declared they knew two or more infected with hepatitis C, it clearly emerges that the infection in question is by no means a marginal occurrence in the current Italian framework, as confirmed by official statistics (7, 58).

Compared to other countries (59–62), Italy does not currently have any specific awareness campaigns on the risk of hepatitis C for university students. Instead, more preventive actions should be promoted through programmes organized in the form of lectures, symposia, conferences, radio programmes, and health talks using all kinds of public platforms and social media, and also through appropriate communication styles that can foster greater interpersonal responsiveness and empathic involvement (63–67). Several studies show that health promotion actions in university settings through active involvement methodologies can produce significant effects in terms of prevention and behavioral change (68–73).

The work should of course be seen in the light of some limitations. One aspect to note is the use of paper-based text scenarios and therefore the failure to evaluate the possibility of using multimedia and digital tools from which young people can be more stimulated. A further limitation was the direct administration of the tools without an articulated discussion of the problems of infection and without having planned group involvement activities through possible peer education actions. Another aspect to be noted is the lack in the experimental design of an efficacy comparison on the intention of diagnostic screening between narrative scenarios with an identification option and a control group that used simple popular and informative material on hepatitis infection.

A future study, which could overcome the above-mentioned limitations, i.e., measuring also the effectiveness of the use of digital tools, including in the protocol a final discussion in the groups in order to collect with qualitative methods a more in-depth feedback on the solicitations perceived during the intervention and to evaluate more carefully the possible changes of attitude and behavioral intentions, adding also a control group in the intervention design, could contribute to a further advancement in the knowledge of the most effective factors and characteristics to be used in the health narrative persuasion. A further line of research could investigate the relationship between frequency of pro-health behaviors in university students and the temporal perspective, since in recent literature there seems to be converging evidence in support of this hypothesis (74–77).

However, we believe that the results of this study, related to hepatitis C prevention, can help policymakers develop communication campaigns aimed at raising awareness and promoting screening according to specific populations, as also suggested by the WHO in the document “Global Health Sector Strategy on Viral Hepatitis, 2016–2021” (17). Better-targeted interventions aimed at enhancing HCV disease risk awareness may ultimately help reduce barriers and increase HCV screening uptake.

## Data Availability Statement

The raw data supporting the conclusions of this article will be made available by the authors, without undue reservation.

## Ethics Statement

The studies involving human participants were reviewed and approved by Institutional Review Board (IRB) of the University of Cassino and Southern Lazio. The patients/participants provided their written informed consent to participate in this study.

## Author Contributions

PD, GV, and SM designed the study. PD, GV, LF, FB, SC, and SM analyzed the data and discussed the results. PD, FB, and SM drafted the manuscript. LF, MF, and FM revised the manuscript. All authors approved the final manuscript. Finally, the authors have agreed to be accountable for all aspects of the manuscript in ensuring that questions related to the accuracy or integrity of any part of it are appropriately investigated and resolved.

## Conflict of Interest

The authors declare that the research was conducted in the absence of any commercial or financial relationships that could be construed as a potential conflict of interest.

## Publisher's Note

All claims expressed in this article are solely those of the authors and do not necessarily represent those of their affiliated organizations, or those of the publisher, the editors and the reviewers. Any product that may be evaluated in this article, or claim that may be made by its manufacturer, is not guaranteed or endorsed by the publisher.
